# Can AM-PAC “6-Clicks” Inpatient Functional Assessment Scores Strengthen Hospital 30-Day Readmission Prevention Strategies?

**DOI:** 10.7759/cureus.14994

**Published:** 2021-05-12

**Authors:** Scott M Arnold, James M Naessens, Kimberly McVeigh, Launia J White, James W Atchison, James Tompkins

**Affiliations:** 1 Physical Medicine and Rehabilitation, Mayo Clinic, Jacksonville, USA; 2 Robert D. and Patricia E. Kern Center for the Science of Health Care Delivery, Mayo Clinic, Rochester, USA; 3 Robert D. and Patricia E. Kern Center for the Science of Health Care Delivery, Mayo Clinic, Jacksonville, USA; 4 Rehabilitation Services, Bayhealth, Dover, USA

**Keywords:** functional status, rehabilitation, readmission, length of stay, discharge planning, am-pac 6-clicks

## Abstract

Background

Prevention of unplanned hospital readmissions remains a priority in the US healthcare sector. Patient functional status has evolved as an important factor in identifying patients at risk for unplanned readmissions and poor predischarge functional performance has been shown to be predictive of increased readmission risk. Yet, patient functional status appears to be underutilized in readmission prediction models.

Methods

To examine the impact of inpatient functional status (mobility and activity performance) on unplanned 30-day hospital readmissions at two tertiary care hospitals, retrospective cohort analysis was performed on electronic health record data from adult inpatients (N = 26,298) having undergone completed functional assessments during their index hospitalization. Primary outcomes were functional assessment scores and unplanned all-cause patient readmission within 30 days following hospital discharge. Secondary analysis stratified the assessment by discharge destination. Functional assessment scores from the Activity Measure for Post-Acute Care (AM-PAC) “6-Clicks” Basic Mobility Short Form or Daily Activity Short Form were extracted along with patient demographics, admission diagnoses, comorbid conditions, and hospital readmission risk score.

Results

Adjusting for age, sex, and comorbidity, lower AM-PAC “6-Clicks” Basic Mobility and Daily Activity scores resulted in higher readmission rates when each score was considered separately. When both scores were considered, only Daily Activity scores were significant.

Conclusion

Patients with lower Basic Mobility and Daily Activity scores are at a higher risk for readmission. The relative importance of AM-PAC “6-Click” scores on short-term readmission depends on discharge destination. Timely identification of patient mobility and activity performance may lead to earlier intervention strategies to reduce readmissions.

## Introduction

With 27% of acute care hospital readmissions estimated to be potentially preventable [1], the focus on minimizing unplanned patient rehospitalization remains a key priority in the US healthcare sector. Rehospitalization within 30 days after discharge from an acute care setting increases risk for negative patient outcomes and contributes to higher healthcare costs [2,3]. When compared to the index hospital admission, the average cost of an unplanned readmission is 15% higher per episode [4].

Growing evidence points to patient functional status as an important determinant of unplanned hospital readmission [5,6]. Several authors have demonstrated poor predischarge performance in functional mobility and self-care abilities to be predictive of increased acute care hospital readmissions [5-8]. Timely identification of key readmission risk factors may facilitate earlier in-hospital intervention strategies [7,9]. 

Notably, the experience of hospitalization itself has been shown to be a risk factor for worsening patient function, especially among the elderly and other vulnerable patients [2,10]. Lack of activity and mobility of hospitalized patients increases the likelihood for secondary medical adverse effects, including hospital-acquired weakness, diminished metabolic function, respiratory compromise, delirium, skin breakdown, venous thromboembolism, and impairment of prehospitalization functional status [11-13]. Inpatient functional status has been underutilized in readmission prediction models as a means for identifying at-risk patients [5,14]. One systematic review of 26 validated readmission risk prediction models identified only two that included patient functional status data; the authors concluded that these two models demonstrated greater accuracy for predicting readmission risk when compared with those without functional status data [14].

The primary aim of this study was to assess the effect that inpatient functional status has on subsequent 30-day readmission. Secondary objectives included exploring differences among those discharged to different post-acute settings and determining whether adding functional status data added greater accuracy to an existing readmission prediction model.

Portions of this study were presented at the 2020 American Physical Therapy Association Combined Sections Meeting in Denver, CO, February 2020, and accepted as an abstract for the 2020 AcademyHealth Annual Research Meeting in Boston, MA, August 2020. Portions of a subset of data from one study site were published in the journal Physical Therapy, February 2021.

## Materials and methods

We conducted a retrospective cohort analysis of unplanned 30-day readmissions and functional status using the Activity Measure for Post-Acute Care (AM-PAC) “6-Clicks” Basic Mobility and Daily Activity Short Forms functional assessment tool [15,16]. The primary analysis was to determine the correlation of functional assessment scores on unplanned all-cause patient readmission within 30 days following hospital discharge. Secondary analyses stratified the assessments by discharge destination and explored whether AM-PAC “6-Clicks” Basic Mobility and Daily Activity scores improved the prediction of 30-day readmission over an existing readmission risk prediction tool. This study was reviewed by the Mayo Clinic Institutional Review Board and determined to be exempt from the requirement for IRB approval (IRB #20-002843).

Outcome measurements

The AM-PAC “6-Clicks” was selected for its reliability and ease of administration, requiring no equipment and only minutes to complete [15,16]. Known as “6-Clicks” due to being composed of six scored patient activity questions, a patient’s “6-Clicks” score falls on a 6- to 24-point scale where a score of 6 represents total functional impairment and a score of 24 represents total absence of impairment. Examiner observation or patient self-report can be used to complete the questionnaire [16]. Two domains of inpatient functional status -- basic mobility and daily activities -- were assessed.

All functional assessments performed in this study were initiated upon provider referral to rehabilitation services for either physical therapy, occupational therapy, or both services. Physical therapists (PTs) measured dimensions of basic mobility functions with the AM-PAC “6-Clicks” Basic Mobility Short Form (turning in bed, sitting on chair, lying to sitting on bed, bed to chair, walking in room, climbing steps) and occupational therapists (OTs) assessed patient ability to perform six activities of daily living with the AM-PAC “6-Clicks” Daily Activity Short Form (dressing upper body, bathing, toileting, dressing lower body, grooming, eating). To determine the “6-Clicks” score, therapists assigned between 1 and 4 points reflecting the level of assistance the patient required in each of the six functional tasks. The AM-PAC “6-Clicks” scoring was captured during each therapy consultation and treatment session and integrated into the patient electronic health record (EHR) as a component of therapist documentation. First and last AM-PAC “6-Clicks” scores were evaluated for patients with multiple assessments within a hospital stay. For patients having only one therapy visit, the initial score was used as both the first and last scores. Not all study patients received both PT and OT consults; therefore, the specific AM-PAC short form employed was dependent on the specific rehabilitation discipline ordered.

Patients and data

This study included all adult patients (N = 26,298) surviving to hospital discharge having undergone completed functional assessments during their hospital stay at two Mayo Clinic hospitals in Phoenix, Arizona and Jacksonville, Florida between January 1, 2016 and December 31, 2017. Patient demographics, diagnoses reflecting reason for hospitalization, Elixhauser comorbidities, Mayo Clinic readmission risk score (RRS), and other relevant hospital information (i.e., emergency admission, clinical service, length of stay, day of week of discharge, surgical status, and discharge destination) were extracted from the EHR [17,18]. The RRS calculator is based primarily on comorbidities, prior utilization, insurance status, and medications [18]. Furthermore, administrative data were extracted for unplanned all-cause patient readmissions within 30 days following the index hospital discharge. Data of unplanned readmissions to facilities outside of our two study hospitals were unavailable for analysis. The AM-PAC “6-Clicks” Basic Mobility and Daily Activity scores obtained contemporaneously were merged for each hospital discharge. For descriptive purposes, we categorized each score into four groups: 6-11, 12-17, 18-23, and 24, where scores below 12 require complete impairment on at least one dimension, a score of 17 or below is equivalent to at least 50% impairment, and a score of 24 implies no impairment [16]. Three hundred thirty-one patients died while being hospitalized and were excluded from the analysis. 

Statistical analysis

We examined the association of AM-PAC “6-Clicks” scores with readmission using t tests and χ^2^ tests for unadjusted scores, identifying the threshold cut point on each score that maximized the odds ratio (OR) for readmission, and multivariate logistic regression models adjusted for patient age, sex, emergency department admission, surgical status, and number of comorbidities. Separate models were also examined by discharge destination (e.g., home, skilled nursing facility [SNF]). A final model included the initial AM-PAC “6-Clicks” scores with the Mayo Clinic RRS to identify if AM-PAC “6-Clicks” Basic Mobility or Daily Activity measures improved accuracy of the currently available readmission risk prediction.

## Results

During the 2-year study period, 55,524 inpatient stays occurred; of these, 26,629 (47.9%) patients received either a Basic Mobility assessment, a Daily Activity assessment or both. A total of 25,628 (46.1%) patients had Basic Mobility assessments, while 17,702 (31.8%) received Daily Activity assessments. As displayed in Table 1, patients with AM-PAC “6-Clicks” assessments had median age of 69, were 52.5% male and closely split between Florida and Arizona hospitalizations. Over half of patients had surgery (54.4%) and about a third were admitted from the emergency room (36.3%). Patients had a mean of 5.2 comorbidities and mean length of stay of 6.5 days (median 4.0).

**Table 1:** Patient Characteristics for Those with Either AM-PAC “6-Clicks” Basic Mobility or Daily Activity Functional Assessments During Hospital Stay at Mayo Clinic Hospital Florida or Mayo Clinic Hospital Arizona, 2016-2017 (n = 26629) SD, standard deviation; ED, emergency department

Characteristics	Value
Age, years	
Mean (SD)	67.2 (15.2)
Median (range)	69.0 (14.0-103.0)
Sex, No. (%)	
Female	12,636 (47.5)
Race, No. (%)	
Missing	329
White	23,239 (88.4)
African American	1,753 (6.7)
Other	1,308 (5.0)
Hospital, No. (%)	
Jacksonville	13,846 (52.0)
Phoenix	12,783 (48.0)
Length of stay, days	
Mean (SD)	6.5 (10.2)
Median (range)	4.0 (1.0-302.0)
ED admission, No. (%)	9,668 (36.3)
Surgery flag, No. (%)	14,477 (54.4)
Elixhauser comorbidity count	
Mean (SD)	5.2 (3.2)
Median (range)	5.0 (0.0-18.0)

Of 26,298 patients discharged alive with AM-PAC “6-Clicks” scores, 3,964 (15.1%) were readmitted: 1,529 (12.4%) from home; 1,169 (17.2%) from home with home health care; 674 (16.4%) from SNF; 536 (28.8%) from IRF; and 56 (11.5%) from other discharge destinations. Readmission rates for all patients in the diagnosis categories used in public reporting through the Centers for Medicare & Medicaid Services Hospital Readmission Reduction Program [19] were examined. Patients with an initial chronic obstructive pulmonary disease diagnosis had the highest readmission rates among targeted diagnosis categories with 19.5% and 20.5% readmission rates for those receiving Basic Mobility and Daily Activity assessments, respectively. Readmission rates for patients with AM-PAC “6-Clicks” scores (Basic Mobility/Daily Activity) among other Hospital Readmission Reduction Program categories included pneumonia (17.9%/18.2%), heart failure (16.2%/17.1%), acute myocardial infarction (12.3%/11.9%), coronary artery bypass graft (7.6%/8.0%), and total hip or knee arthroplasty (2.5%/2.4%).

Unadjusted AM-PAC “6-Clicks” Basic Mobility and Daily Activity scores were significantly lower for readmitted patients (p < 0.001). The figure shows the 30-day readmission rate by score group for Basic Mobility and Daily Activity scores. In general, patients with lower scores were more likely to be readmitted; however, patients at the lowest levels of functioning (6-11) on either score were slightly less likely to be readmitted than patients with scores from 12 to 17. For Basic Mobility, a threshold analysis suggested that a score lower than 17 maximized odds of readmission compared to scores higher than 17 (OR, 1.19; 95% confidence interval [CI], 1.13-1.24). Threshold analysis for Daily Activity found that a score lower than 19 maximized readmissions compared to scores higher than 19 (OR, 1.30; 95% CI, 1.25-1.35). When adjusting for age, sex, comorbidity, surgery, and emergency department admission and considering Basic Mobility and Daily Activity scores individually, lower scores increased readmissions (Basic Mobility OR, 0.99, p = 0.008; Daily Activity OR, 0.98, p < 0.001; C statistic, 0.679 for each model). When analysis was adjusted and both scores were considered simultaneously, patients with lower Daily Activity scores were still more likely to be readmitted (Table 2: Basic Mobility OR, 1.0, p = 0.84; Daily Activity OR, 0.98, p = 0.01; C statistic, 0.680).

**Figure 1:**
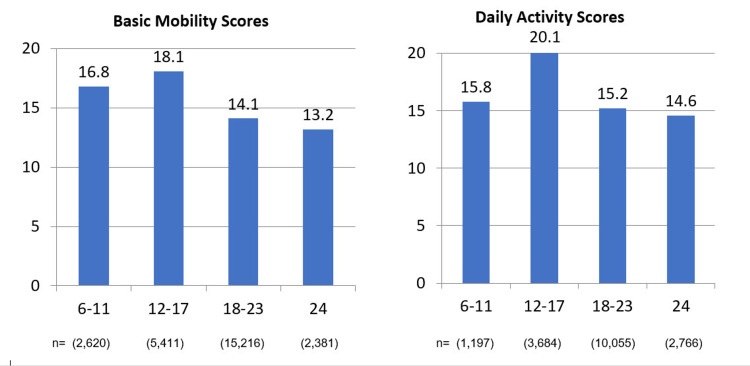
30-day Readmission Rate by Unadjusted 6-click Scores

**Table 2:** Results of Multivariate Logistic Models Examining the Association of Basic Mobility and Daily Activity with Readmission, Incorporating Patient Age, Sex, ED Admission, Surgery, and Comorbidity ED, emergency department ^a^Patients without “6-Clicks” Daily Activity assessments (i.e., no occupational therapy visit) were assigned scores of 24 and had this flag set to 1, patients with assessments had this flag set to 0

Effect	Odds Ratio (95% CI)	p-Value
Age (10-year increments)	0.89 (0.86-0.91)	<0.001
Sex, male	1.16 (1.08-1.24)	<0.001
ED admission	1.15 (1.06-1.24)	<0.001
Surgery	0.86 (0.80-0.94)	<0.001
Elixhauser count	1.18 (1.17-1.20)	<0.001
Basic Mobility score	1.00 (0.99-1.01)	0.84
Daily Activity score	0.98 (0.97-1.00)	0.010
Daily Activity score missing flag^a^	0.93 (0.84-1.02)	0.14

When examining these models within specific discharge destinations (Table 3), lower initial Daily Activity scores were associated with more readmissions for patients sent home without additional support, and lower initial Basic Mobility scores were associated with more readmissions for patients sent to SNF, while higher initial Basic Mobility scores were associated with more readmissions for patients sent home with home healthcare services. When adding the Basic Mobility and Daily Activity scores to a model including all adjustment factors and the RRS, higher Basic Mobility scores were associated with more readmissions. 

**Table 3:** Multivariate Logistic Models Examining the Association of Basic Mobility and Daily Activity with Readmission Incorporating Patient Age, Sex, Comorbidity, Surgery, and ED Admission by Discharge Status Category ED, emergency department; SNF, skilled nursing facility

Discharge Destination	Effect	Odds Ratio (95% CI)	p-Value	c-stat
Home	Age (10-year increments)	0.84 (0.81, 0.87)	<0.001	
	Male	1.13 (1.01, 1.27)	0.04	
	ED admission	1.15 (1.00, 1.32)	0.04	
	Surgery	0.77 (0.67, 0.88)	<0.001	
	Elixhauser count	1.21 (1.19, 1.23)	<0.001	
	Basic Mobility score	1.01 (0.99, 1.03)	0.50	
	Daily Activity score	0.97 (0.95, 0.99)	0.01	
	Daily Activity score missing flag	1.04 (0.90, 1.19)	0.63	0.705
Home with home health care	Age (10-year increments)	0.88 (0.84, 0.92)	<0.001	
	Male	1.07 (0.94, 1.22)	0.31	
	ED admission	1.19 (1.02, 1.38)	0.02	
	Surgery	0.83 (0.71, 0.97)	0.02	
	Elixhauser count	1.18 (1.16, 1.21)	<0.001	
	Basic Mobility score	1.02 (1.00, 1.04)	0.04	
	Daily Activity score	0.99 (0.97, 1.02)	0.51	
	Daily Activity score missing flag	1.05 (0.88, 1.26)	0.59	0.688
SNF	Age (10-year increments)	0.90 (0.84, 0.98)	0.01	
	Male	1.39 (1.17, 1.65)	<0.001	
	ED admission	1.25 (1.04, 1.51)	0.02	
	Surgery	0.80 (0.66, 0.97)	0.02	
	Elixhauser count	1.16 (1.13, 1.20)	<0.001	
	Basic Mobility score	0.97 (0.95, 1.00)	0.04	
	Daily Activity score	1.02 (0.99, 1.05)	0.11	
	Daily Activity score missing flag	0.74 (0.55, 1.01)	0.06	0.668
Rehab	Age (10-year increments)	0.95 (0.88, 1.02)	0.17	
	Male	1.25 (1.01, 1.54)	0.04	
	ED admission	0.97 (0.78, 1.21)	0.78	
	Surgery	1.26 (1.01, 1.57)	0.05	
	Elixhauser count	1.15 (1.11, 1.19)	<0.001	
	Basic Mobility score	1.01 (0.98, 1.05)	0.41	
	Daily Activity score	1.01 (0.98, 1.04)	0.49	
	Daily Activity score missing flag	0.39 (0.23, 0.66)	<0.001	0.642

## Discussion

Based on previous reports, it was anticipated that patients with impaired functional status would be readmitted at a higher rate. This multisite study demonstrated that lower AM-PAC “6-Clicks” patient functional assessment scores were associated with increased 30-day readmission rates regardless of discharge destination. Cut points to maximize odds of readmission were determined to be scores less than “17” for Basic Mobility and below “19” for Daily Activity. When considered separately in models adjusted for important patient and hospitalization factors, lower Basic Mobility and Daily Activity scores were significant predictors (Basic Mobility OR, 0.99, p = 0.008; Daily Activity OR, 0.98, p < 0.001). However, when both scores were included, lower Daily Activity scores were associated with more readmissions, but Basic Mobility scores provided no further influence. This is likely due to the high correlation between the two measures, but it does appear that different aspects of function may be more influential on readmission risk in different settings and warrant further study. When looking at adjusted models including both scores for specific discharge destinations, lower Daily Activity was associated with more readmissions among patients going home without additional services, lower Basic Mobility was associated with more readmissions for patients going to SNF, and higher Basic Mobility was associated with more readmissions for patients going home with home healthcare services. While seemingly paradoxical, this last finding may have several explanations. The home healthcare service referral at discharge may be unrelated to mobility impairments. A patient may need home infusion to treat a chronic infection, for example, but can mobilize independently, and therefore, may have a Basic Mobility score of “24” (no functional impairment), while still be at relatively high risk of readmission due to an underlying medical issue. Unfortunately, the specific health services provided in the home could not be determined for this study.

Considering the association between functional status and subsequent readmission, some authors have advocated for expanding the assessment of functional status to a greater percentage of hospitalized patients [20]. While our study used functional assessments collected by rehabilitation therapists, employing a quick and easily administered tool such as the AM-PAC “6 Clicks” may allow for a greater percentage of patients to be screened by other staff if current PT/OT numbers are limited; in our own study, less than half (47%) of the hospitalized patients received orders for PT and/or OT. Reports of neuroscience nursing staff at Johns Hopkins Hospital performing patient AM-PAC “6-Clicks” Basic Mobility assessments have been described, with interrater reliability comparable to that of PTs, ranging from 0.91 (95% CI, 0.86-0.95) to 0.96 (95% CI, 0.93-0.98) [21]. Expanding assessment of patient functional status by other staff may offer the advantage of greater proportion of patient assessments due to comparatively larger staffing numbers combined with correspondingly greater amount of time spent in direct patient contact. Expansion of functional assessment screening to other staff may also support current interest in a culture of mobility that emphasizes early activity and mobility for inpatients during their acute hospital stay. Structured mobility programs have shown reductions in readmissions, patient falls, and skin breakdown [22,23]. Additionally, proponents of systematic functional assessment point out the benefit of improving communications by having common terminology for describing patient functional ability across interdisciplinary care team members (e.g., physicians, advanced practice providers, nurses, case managers, PTs, and OTs) and discharge destination settings [21,23]. Subsequent referrals to PT and OT can be made as functional deficits are identified.

As specialists in recognizing and managing functional impairment, PTs and OTs play important roles in making post-hospital transition recommendations for appropriate disposition and levels of continued rehabilitation services based on these impairments [7,9,24,25]. A study of discharge planning and readmissions determined that inclusion of a PT on the interdisciplinary discharge team impacted 30-day readmission rates; odds for readmission for patients from the group having no PT on the interdisciplinary team were 3.78 times greater than patients from the group with a PT on the interdisciplinary discharge team [26]. Rehabilitation therapists’ recommendations are an important component of a complex care transition process, characterized by substantial variability among numerous determinants (e.g., insurance benefits, home environment, patient self-care capacity, available caregivers, access to transportation, socioeconomic considerations, patient/family preferences, and facility care transition practices) [25,27]. While these variables are often outside the control of the patient, provider, or discharging facility to alter, predischarge functional status represents an important modifiable risk factor involved in early readmission. Using functional status to identify patients at risk for unplanned readmission may enable strategies for earlier resource allocation, such as rehabilitation interventions, to minimize functional decline while in the acute care setting [7,9]. Earlier intervention also holds the potential for discharging patients to lesser levels of post-acute care, while discordance between therapist recommendations with actual discharge destinations and levels of service have been reported to increase the risk of readmission [25]. In 2010, Smith et al. examined actual versus recommended discharge destinations and services made by 40 PTs for 762 patients discharged from a large academic medical center [25]. The probability of hospital readmission was 2.89 times greater when the PT’s recommendations for post-acute care services were not implemented (OR, 2.89, 95% CI, 1.57-5.30) [25]. Additionally, the timing of the onset of discharge planning during a patient’s hospital stay has been shown to impact readmissions, with earlier strategies associated with reduced readmissions, lower mortality, and improved quality of life [28]. Initial AM-PAC “6-Clicks” scores alone have been shown to accurately predict hospital discharge destination and may allow discharge planners additional data to initiate post-acute care transition planning earlier in the index admission [29].

Study limitations

This retroactive study has limitations; data collection was restricted to two urban academic hospitals. Not all hospitalized patients were assessed for functional status while in the hospital. Results of the impact of functional status on readmission may, therefore, be biased in our multivariate modeling. We did incorporate a missing data indicator for those with basic mobility assessments but not daily activity assessments to distinguish between those with no functional impairments and those not assessed. However, this adjustment will not remove all potential bias. Furthermore, no attempt was made to capture and report patient cognitive status or social determinants of health; thus, their effects on patient readmission are unknown. No data on patient insurance status were collected. Likewise, no data were included on patient readmissions to other medical facilities outside our own; therefore, the actual readmission rate of discharged patients may have been underreported. Rehabilitation therapist recommendations for post-acute care discharge destination versus patient actual destination and services were not evaluated; hence, effects of potential mismatch between recommended and actual post-acute care settings and services upon odds of subsequent readmissions are undetermined.

## Conclusions

Our study confirms that lower AM-PAC “6-Clicks” functional assessment scores are significantly associated with increased 30-day hospital readmissions. The AM-PAC “6-Clicks” assessment offers a simple and clinically efficient tool for clinicians to quickly recognize and uniformly quantify functional impairment in the inpatient setting. Early identification of limited patient functional status may help assist clinicians to focus on predischarge rehabilitation intervention strategies aimed at reducing the likelihood of hospital readmission among identified at-risk patients. Expanding functional assessment screening to inpatients not receiving PT or OT warrants further study.
